# Privacy-preserving semi-parallel logistic regression training with fully homomorphic encryption

**DOI:** 10.1186/s12920-020-0723-0

**Published:** 2020-07-21

**Authors:** Sergiu Carpov, Nicolas Gama, Mariya Georgieva, Juan Ramon Troncoso-Pastoriza

**Affiliations:** 1 CEA, LIST, Point Courier 172, Gif-sur-Yvette cedex, 91191 France; 2 Inpher, Innovation Park A, Lausanne, CH-1015 Switzerland; 3 EPFL, Route Cantonal, Lausanne, CH-1015 Switzerland

**Keywords:** Fully homomorphic encryption, Logistic regression, Genome privacy, Genome-wide association study

## Abstract

**Background:**

Privacy-preserving computations on genomic data, and more generally on medical data, is a critical path technology for innovative, life-saving research to positively and equally impact the global population. It enables medical research algorithms to be securely deployed in the cloud because operations on encrypted genomic databases are conducted without revealing any individual genomes. Methods for secure computation have shown significant performance improvements over the last several years. However, it is still challenging to apply them on large biomedical datasets.

**Methods:**

The HE Track of iDash 2018 competition focused on solving an important problem in practical machine learning scenarios, where a data analyst that has trained a regression model (both linear and logistic) with a certain set of features, attempts to find all features in an encrypted database that will improve the quality of the model. Our solution is based on the hybrid framework Chimera that allows for switching between different families of fully homomorphic schemes, namely *TFHE* and *HEAAN*.

**Results:**

Our solution is one of the finalist of Track 2 of iDash 2018 competition. Among the submitted solutions, ours is the only bootstrapped approach that can be applied for different sets of parameters without re-encrypting the genomic database, making it practical for real-world applications.

**Conclusions:**

This is the first step towards the more general feature selection problem across large encrypted databases.

## Background

The advent of next generation sequencing and the progressive reduction of costs in sequencing processes result in an increasing amount of available genomic data, which is essential for better modeling the relation between genotypic traits, predisposition to diseases, response to treatments, effect of drugs, and, in general, for achieving more accurate models that enable personalized and precision medicine. While machine learning computations on large scale genomic data present obvious outsourcing needs and can benefit from Cloud services, the high sensitivity of genomic data and the impossibility of properly anonymizing it [1, 2] call for effective protection methods that enable accurate and efficient computation without leaking information about the individual genomic sequences to untrusted cloud service providers. In order to become feasible and usable for the purpose of personalized medicine, these protection mechanisms must optimize the trade-off between the accuracy of the results, the efficiency of the computation, and the security level.

In this context, the iDASH Privacy and Security Workshop has joined together experts on privacy enhancing techniques, applied cryptography and secure computation to design and implement secure and privacy-preserving solutions to fundamental genomics and bioinformatics problems. iDASH has pushed the state of the art on practical secure computation by organizing a world-wide competition to evaluate the most advanced techniques in the field. In particular, in its 2017 and 2018 editions, iDASH featured a track focused on training logistic regression models on encrypted genomic datasets, by relying on Homomorphic Encryption (HE), which enables certain operations (additions and/or multiplications) to be performed on encrypted ciphertexts without the need to decrypt them first.

Linear and logistic regressions are one of the most common and versatile machine learning tools used in genomic studies. These are the core of Genome Wide-Association Studies (GWAS), and its privacy-preserving implementation represents a first step towards effective and efficient outsourced machine learning on genomic data.

During the last years, there have been numerous approaches to implement these operations securely on medical data; these involve, on the one hand, (a) distributed settings where two or more parties collectively compute a function such as a linear or logistic regression, by applying technologies such as garbled circuits [3], in settings limited to two parties, or secret sharing and multiparty computation [4], through interactive protocols; these solutions require non-colluding computing parties and heavily rely on communication between them; on the other hand, (b) outsourced scenarios move the bulk of the computation to an untrusted third party in a non-interactive setting, either relying on trusted hardware such as Intel SGX [5–8], therefore requiring some degree of trust on the hardware manufacturer, or homomorphic encryption [9–14]. The latter does not need any assumption on the hardware, as it is solely based on the cryptographic guarantees of the used cryptosystems, so it can be seen as the most promising approach for outsourcing medical computations.

In 2016, Aono et al. [9] proposed a solution for training a logistic regression based on additive homomorphic encryption, which requires the client to precompute some intermediate values in order to account for the limited range of operations (additions) supported under encryption. Afterwards, most of the finalists of the HE track in iDASH 2017 leveraged input packing and somewhat homomorphic cryptosystems (SHE), enabling both encrypted additions and a limited number of encrypted products, to implement the basic logistic regression block; i.e., a Gradient descent algorithm with an approximated Sigmoid function on an encrypted matrix of input data; the sought output is the vector of regression coefficients. Bonte et al. [12] implemented one iteration of a simplified fixed Hessian method with the Fan-Vercauteren (FV) SHE cryptosystem; Kim et al. [11] employed a Nesterov’s accelerated Gradient descent algorithm with the *HEAAN* SHE cryptosystem, which supports homomorphic rescaling and approximate arithmetic; Chen et al. [13] implemented 1-bit Gradient descent with a modified FV cryptosystem featuring rescaling and bootstrapping, but the used bootstrapping introduces a notable performance penalty. In 2018, in parallel with our work, Crawford et al. [10] introduced a fully homomorphic encryption (FHE)-based method for the same problem that relies on the BGV cryptosystem, and requires to solve a linear system of equations in the client after decryption; despite the many optimizations used in the work, the bootstrapping takes 75% of the computation time, and this is still notably higher than the previous SHE-based solutions.

The HE track in iDASH 2018 has evolved in complexity, targeting a more advanced semi-parallel logistic regression algorithm that outputs the p-values of the trained regression estimates. In this paper, we propose a solution to semi-parallel logistic regression on encrypted genomic data based on fully homomorphic encryption, that leverages on a novel framework, Chimera [15], to (a) seamlessly switch between different Ring-LWE-based ciphertext forms, therefore combining the advantages of each of the existing Ring-LWE-based cryptosystems to perform each of the steps of the process in a more efficient way, and (b) is generic, in such a way that it can cope with arbitrary input sizes (number of covariates, number of records, and number of genomic variants), and (c) features two configurations depending on the sought trade-off between accuracy and confidentiality.

## Methods

**Notation**

We denote by $\mathbb {T}$ the real Torus $\mathbb {R}/\mathbb {Z}$, the set of real numbers modulo 1. We denote by $\mathbb {Z}_{N}[\!X]=\mathbb {Z}[X]/(X^{N}+1)$ the ring of polynomials with integer coefficients modulo *X*^*N*^+1. Respectively, $\mathbb {R}_{N}[\!X]=\mathbb {R}[\!X]/(X^{N}+1)$ is the ring of real polynomials modulo *X*^*N*^+1. We denote the $\mathbb {Z}_{N}[\!X]$-module $\mathbb {T}_{N}[\!X]= \mathbb {R}_{N}[\!X] / \mathbb {Z}_{N}[\!X] $ (a.k.a $\mathbb {R}[\!X] \bmod X^{N}+1 \bmod 1$). We denote also $\mathbb {B}_{N}[\!X]$ as the subset of $\mathbb {Z}_{N}[\!X]$ with binary coefficients.

We provide now a brief description of the two Ring-LWE homomorphic schemes used in this work, namely *TFHE* [16, 17] and *HEAAN* [18] (a.k.a CKKS), both enabling error-tolerant decryption functions, and hence approximated arithmetic, and we present then the Chimera framework [15] unifying both.

*TFHE* (Torus Fully Homomorphic Encryption) [16] defines messages and ciphertexts over the torus modulo 1 ($\mathbb {T}=\mathbb {R}/\mathbb {Z})$, and keeps track of the noise standard deviation *α*≪1, a dynamic parameter that changes after each operation. Therefore, plaintexts have *ℓ*=− log2(*α*) fractional bits of precision. *TFHE* can represent three plaintext spaces, with various morphisms or actions to switch between them:

- *TLWE* encodes individual (continuous) messages over the torus $\mathbb {T}$;
- *TRLWE* encodes (continuous) messages over $\mathbb {R}[X] \bmod (X^{N} + 1) \bmod 1$, which can be viewed as the packing of *N* individual coefficients;
- *TRGSW* encodes integer polynomials in $\mathbb {Z}_{N}[X]$ with bounded norm.

We describe below the main algorithms that are used for the *TFHE* with *TRLWE* encryption scheme, considering a security parameter *λ*=128, and a minimal noise standard deviation *α*; these parameters implicitly define a minimal key size *N*≈ max(256,32*α*) (see Section 6 of [17]). KeyGen/Phase: A uniformly random binary key $s\in \mathbb {B}_{N}[X]$, this implicitly defines the secret *phase* function $\varphi : \mathbb {T}_{N}[\!X]^{2} \to \mathbb {T}_{N}[\!X], (a,b)\mapsto (b-sa)$. Encrypt (*μ*,**s**,*α*): Pick a uniformly random $a\in \mathbb {T}_{N}[\!X]$, and a small Gaussian error *e* from $\mathbb {T}_{N}[\!X]$ with standard deviation *α*, and return (*a*,*s*.*a*+*μ*+*e*). DecryptApprox(*c*,*s*): Return *φ*_*s*_(*c*), which is close to the actual message. The error (the distance between the plaintext and the output of DecryptApprox(*c*,*s*)) has the same order of magnitude as *α*. Decrypt($c, s, \mathcal {M}$): Round *φ*_*s*_(*c*) to the nearest point in $\mathcal {M}$.

Arithmetic operations supported by *TFHE* are the addition and the multiplication of plaintext messages. We differentiate 2 types of multiplication: (i) internal – multiply 2 *TRLWE* samples and (ii) external – multiply a *TRGSW* and a *TRLWE* sample. The external multiplication is faster and the noise increase is smaller. The public key switch operation allows to evaluate a linear function with integer coefficients over *TLWE* or *TRLWE* input samples. A private key switch allows to hide the integer coefficients. The sample extract operation allows to obtain a *TLWE* sample that encodes the *i*-th polynomial coefficient of an input *TRLWE* sample with at most the same noise variance or amplitude.

Bootstrapping traditionally evaluates the rounding function (homomorphic decryption) on the encrypted plaintext, in order to refresh a noisy ciphertext $\mathfrak {c}$. The *gate bootstrapping* in *TFHE* can refresh a noisy *TLWE* ciphertext $\mathfrak {c}$, but it can be more general, by also changing the plaintext space; i.e., the gate bootstrapping algorithm allows to evaluate any pointwise defined negacyclic function $f:\mathbb {T} \rightarrow \mathbb {T}$ to the phase of a *TLWE* sample.

Finally, it is worth noting that any *TLWE*, *TRLWE*, *TRGSW* ciphertext, bootstrapping key or keyswitching key given at a given precision, can always be rounded and truncated to match the current (lower) precision *α*. Whenever *α* varies (e.g. increases after each multiplication, or decreases after a bootstrapping), we always use the last keyswitching and bootstrapping operation to switch to a new encryption key whose entropy is as close as possible to the lower bound *N*≈ max(256,32*α*) from the security estimates.

*HEAAN* [18] also supports approximate arithmetic; its message space is the set of small-norm polynomials with coefficients in $\mathcal {R}_{q}$. The least significant bits of a message *μ* are considered as noise, and only its most significant bits are required to have a correct decryption. A *HEAAN* ciphertext is a Ring-LWE tuple $(a,b)\in \mathcal {R}^{2}_{q}$, where *a* is uniformly random in $\mathcal {R}_{q}$, and *b* is close to *a*·*s*+*μ*, up to a Gaussian error of small amplitude. Plaintexts and ciphertexts share the same space, and homomorphic multiplication of two ciphertexts involves a relinearization with a keyswitch operation, followed by a modulus-rescaling operation that rescales both plaintext and ciphertext; this helps managing not only the noise growth but also the plaintext growth, keeping a constant upper bound on the message.

### Chimera: unifying *HEAAN* and *TFHE*

As shown in [15], both *HEAAN* and *TFHE* use the same ciphertext space (up to rescaling by a factor *q*), and the *TFHE* notion of phase can be extended to *HEAAN*. The Chimera framework interprets the plaintext spaces as subsets of the same module $\mathbb {T}_{N}[X]$, and uses the distance function on the torus to quantify the transformation error; then, both schemes use the same ciphertext space $\mathbb {T}_{N}[X]^{2}$, the same key space $\mathbb {B}_{N}[X]$ and the same phase function *φ*_*s*_(*a*,*b*)=*b*−*s*·*a*. In this framework, decryption finds two definitions: the first one, common to *HEAAN* and *TFHE*, considers that the phase is always close (within distance <*α*) to the actual message and is a good enough approximation thereof. Then, accumulated errors are not corrected by the cryptosystem (but rather by the numerical stability of the homomorphically evaluated algorithm). The second decryption, unique to *TFHE*, restricts the valid message space to a discrete subset of $\mathbb {T}_{N}[X]$ with a packing radius ≥*α*. Then, the exact message is recovered by rounding the phase to the closest valid message.

In this unified plaintext space $\mathbb {T}_{N}[X]$, it is important to preserve the notion of *user-side slots*, which corresponds to the way the end-user actually employs the schemes. Following *HEAAN* formulation, homomorphic operations are presented as *N*/2 SIMD (Single Instruction Multiple Data) slots containing complex numbers in fixed-point representation with the same public exponent and the same precision. By interpolating the complex roots of *X*^*N*^+1, the native plaintext can be mapped to small polynomials of $\mathbb {T}_{N}[X]$. Hence, it is possible to represent (pack) a plaintext message either with *slot packing* (enabling component-wise products) or with *coefficient packing* (enabling convolution products), and both representations are related to each-other by a homomorphic linear transform.

Finally, Chimera also preserves the notion of levels common to *TFHE* and *HEAAN*: the level *L*≥0 bounds the ratio between the ciphertext modulus and the native plaintext modulus, and therefore the number of homomorphic operations supported by the ciphertext. Each homomorphic product reduces the level of the resulting ciphertext; when the level 0 is reached, the ciphertext must be bootstrapped to continue operating on it.

### Semi-parallel logistic regression and our simplification

The HE track in iDASH 2018 consists in executing a semi-parallel logistic regression [19] with encrypted phenotypic and genotypic features; the former are represented as a covariate matrix *X* (*k*+1 covariates ×*n* patients), and the latter as a binary SNP matrix *S* (*n* patients ×*m* SNPs). The original method in [19] is sketched in Algorithm 1; it comprises two parts: first, it builds a logistic regression model using only phenotype features (i.e. covariates matrix *X*) through an iterative process (i.e. gradient descent); afterwards, it updates this model with genotype features (matrix *S*). The outputs of the algorithm are the *p*-values of the estimates after training the model. The obtained acceleration factor (compared to doing individual logistic regressions) comes mainly from the fact that the second part is performed once and includes all genotype features *S*.


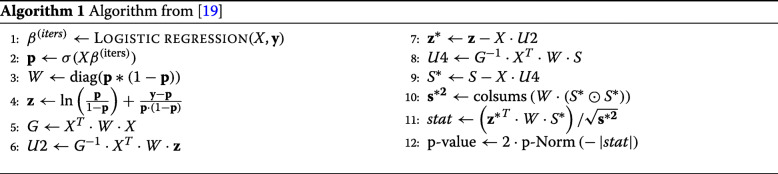


In this work we have further simplified the approach proposed in [19], resulting in Algorithm 2. First, let *E* be the subspan generated by the columns of $X'=\sqrt {W}X$, *z*^′^ be the vector $\sqrt {W}z$, *z*^′∗^ be the vector $\sqrt {W}z^{*}$, *S*^′^ be the matrix $\sqrt {W}S$, and *S*^′∗^ be the matrix $\sqrt {W}S^{*}$. With this change of variable, Line 7 and 9 result: *z*^′∗^=*z*^′^−*X*^′^(*X*^′*T*^*X*^′^)^−1^*X*^′*T*^*z*^′^*S*^′∗^=*S*^′^−*X*^′^(*X*^′*T*^*X*^′^)^−1^*X*^′*T*^*S*^′^.In other words, *z*^′∗^ and *z*^′∗^ are the orthogonal projection of *z*^′^ and *S*^′^ over *E*^⊥^. Note that by definition, $z^{\prime }=X'\beta + \sqrt {W}^{-1}(y-p)$. In this sum, the first operand is in *E* by construction, and we verify that the second operand is in *E*^⊥^. Indeed, the dot product $X^{\prime T}.\sqrt {W}^{-1}(y-p)=X(y-p)$ is the gradient of the cost function of the logistic regression, and is null at the point of convergence. Therefore, the projection *z*^′∗^ is equal to $\sqrt {W}^{-1}(y-p)$, and the numerator of the stat (line 11) simplifies to the FHE-friendly expression: *z*^∗*T*^*WS*^∗^=*z*^′∗*T*^*S*^′∗^=(*y*−*p*)^*T*^.*S*

For the denominator of the stat, we note that for all *j*∈[1,*m*], $\mathbf {s}_{j}^{*2}=S_{j}^{*T}WS_{j}^{*}=\left \Vert {S_{j}^{\prime *}}\right \Vert ^{2}$ where $S_{j}^{\prime *}$ is the *j*-th column of *S*^′∗^. By definition of the orthogonal projection, we therefore have $\mathbf {s}_{j}^{*2}=\left \Vert {S_{j}^{\prime }}\right \Vert ^{2}-\left \Vert {\pi _{E}(S_{j}^{\prime })}\right \Vert ^{2}$ where $\pi _{E}(S_{j}^{\prime })$ is the orthogonal projection of $S_{j}^{\prime }$ on *E*. Namely, if we call *A*=*X*^′*T*^*S*^′^=*X*^*T*^*WS*, then $\left \Vert {\pi _{E}(S_{j}^{\prime })}\right \Vert ^{2}=A_{j}^{T} G^{-1} A_{j}$. Therefore, once we have precomputed *A* and *G*^−1^, line 10 can be simplified as: **s**^∗2^=colsums(*W*·(*S*⊙*S*))−colsums(*A*⊙*G*^−1^*A*), and any other intermediate variable that does not appear in this formula can be removed from the pseudocode. As a bonus, for binary valued matrices *S*, (*S*⊙*S*) is equal to *S*, and due to the geometric interpretation of the logistic regression and the projections, the input matrix *X* can be replaced by any basis of the same vector span without affecting the final result.


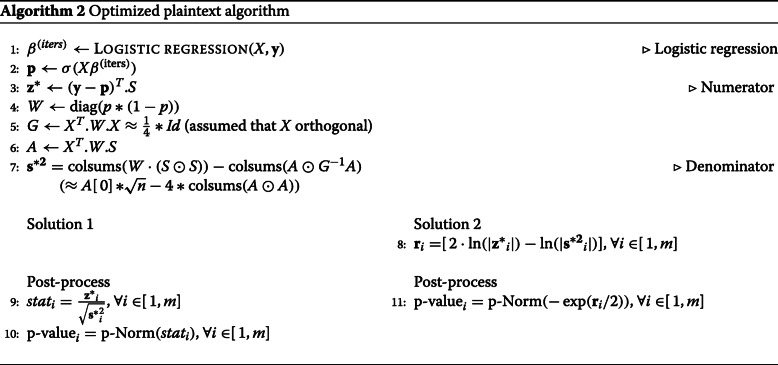


The algorithm must also be transformed to use fixed-point data type instead of floating-point, due to the homomorphic encryption libraries we use. Hence, input data must be carefully scaled so that no overflow happens during the execution. We have performed simulations with the optimized algorithm in order to determine the ranges of intermediary variables. Table 1 depicts the obtained simulation results. These ranges are used for scaling input data. The inverse sigmoid function applied on the probability vector **p** gives us an input range −1.6…1.8 of the sigmoid function; this input corresponds to elements of vector *X*·*β*^*iters*^. This range is extended to −4…4 (*σ*_*min*_…*σ*_*max*_) to allow a margin of error. By mapping this range to the plaintext space of Torus based homomorphic libraries, the scaling factor $\frac {1}{16}$ of *X*·*β*^*iters*^ is obtained. Note that only the $-\frac {1}{4}\ldots \frac {1}{4}$ part of Torus is used in our computations because of the negacyclic property of functions evaluated by the *TFHE* bootstrapping procedure. Propagating the range of *X*·*β*^*iters*^ backward and forward in Algorithm 2 the scaling factors for other variables (including input data) are obtained. An automatic tool, or semi-automatic, for computing fixed-point range and scale for algorithm variables together with respective mapping to encryption scheme parameters would have been very helpful. We leave this research and development issue for a future work.

**Table 1:** Data ranges of intermediary variables in the plaintext Algorithm 2

Variable	avg	stdev	min	max
**p**	0.440816	0.0975715	0.176397	0.853487
*W*	0.236977	0.0201871	0.125047	0.25
z*	-3.33092	7.36068	-30.9426	31.2008
G	0.0577846	0.0953495	-0.011997	0.236977
A	0.0621965	0.301255	-0.317312	2.236
s^∗2^	2.44243	4.11085	0.111961	14.5044
r_i_	0.200039	1.84459	-13.7207	4.36158
*p*-value	0.310218	0.24083	0	0.999163

The average, standard deviation, minimum and maximum statistics are shown

### FHE algorithm

The proposed solution is split into 3 sequentially executed parts, which are implemented using different homomorphic encryption techniques and libraries. As shown in Algorithm 2, our solution features two options, depending on the sought trade-off between confidentiality and accuracy. The first solution outputs both numerator and denominator of the stats, while the second solution outputs only the quotient, which is equivalent to the p-value. We explain in detail each part and the encryptions of input data in the next sub-sections.

#### Step1 – logistic regression

Algorithm 3 illustrates a more explicit version of the implemented logistic regression algorithm. We have used the *TFHE* library [16] to homomorphically execute this algorithm.

##### Input data encryption.

Input covariates matrix *X* and outcome vector *y* are encrypted using different encoding and HE scheme types. Each column of the covariate matrix is encrypted in a *TRGSW* ciphertext. A total of *k* ciphertexts are used for matrix *X*. The outcome vector is encrypted in a single *TRLWE* ciphertext. Besides these encryptions we use additional ones used by the bootstrapping procedure. For the sake of simplicity we omit input data scaling factors, mentioned in previous subsection, from our discourse. In what follows we describe the encoding we use:

- *j*-th column of matrix *X* is coefficient packed into a polynomial $P_{X_{.,j}}(Z) = \sum _{i=0}^{n-1} X_{i,j} \cdot Z^{i}$ and encrypted in a *TRGSW* ciphertext (L1 and L2)
- vector *y* (scaled by step *α*) is coefficient packed in reverse order into a polynomial $P_{y}(Z) = \alpha \cdot \sum _{i=0}^{n-1} y_{n-i-1} \cdot Z^{i}$ and encrypted in a L2 *TRLWE* ciphertext
- test polynomials for gate bootstrapping encode sigmoid function (defined over the range [*σ*_min_,*σ*_max_] and discretized on *d*=⌊*N*/*k*⌋−1 levels) multiplied by matrix *X* rows and encrypted as L2 *TRLWE* ciphertexts:
  - ${TP}_{i}(Z) = \sum _{q=0}^{d} \alpha \cdot \sigma \left (\sigma _{min} + \frac {q}{d}\cdot \left (\sigma _{max}-\sigma _{min}\right) \right) \sum _{j=0}^{k-1} X_{i,j} \cdot Z^{q\cdot k + j - N/2} $

##### *TFHE* implementation.

Subsequently we explain how each operation in Algorithm 3 is performed using the *TFHE* library.


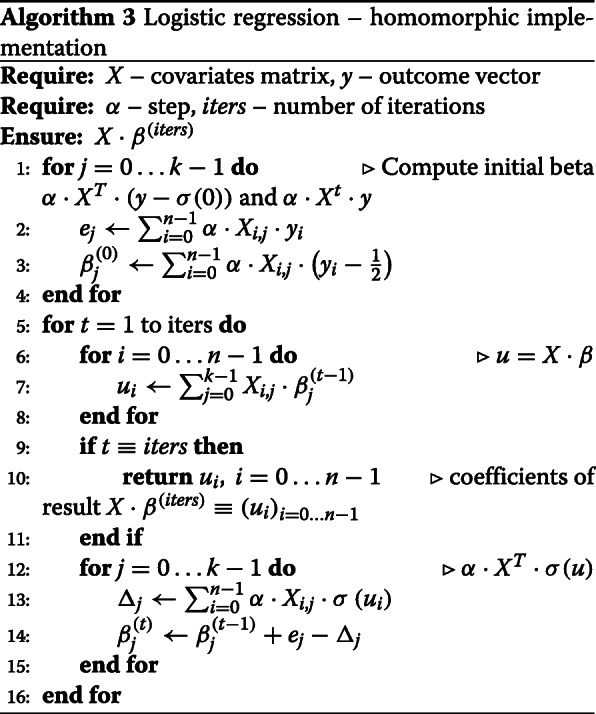


**Line 2.** Firstly, L2 encryptions of matrix *X* column ($P_{X_{.,j}}$) and outcome vector (*P*_*y*_) are multiplied together (external product). This results in an encryption of $\sum _{i=0}^{n-1} \alpha \cdot X_{i,j} \cdot y_{i} \cdot Z^{n-1} + \ldots $. The (*n*−1)-th coefficient of this polynomial is the scalar value *e*_*j*_. The coefficient extraction procedure is used to obtain a L2 *TLWE* encryption of *e*_*j*_.

**Line 3.** This operation is similar to the previous one except that: (i) beforehand plaintext $\alpha \cdot \sum _{i} \frac {1}{2}\cdot Z^{i}$ (note that $ \frac {1}{2} \equiv \sigma (0)$) is subtracted from the encryption of *P*_*y*_ and (ii) a key-switching procedure is used to obtain a L1 *TRLWE* encrypting $\beta ^{(0)}_{j}$ from the L2 *TLWE* sample after the coefficient extraction. A copy of L2 *TLWE* encryption of $\beta ^{(0)}_{j}$ is kept for update performed in algorithm line 14.

**Line 7.** L1 *TRLWE* encryption of $\beta ^{(t-1)}_{j}$ (from previous iteration) and L1 *TRGSW* encryption of $P_{X_{.,j}}$ are multiplied together for all *j*=0…*k*−1. Encryptions of polynomials $\sum _{i} X_{i,j}\cdot \beta ^{(t-1)}_{j} \cdot Z^{i}$ are obtained. These L1 *TRLWE* ciphertexts are summed-up and an encryption of polynomial $\sum _{i} \sum _{j} X_{i,j}\cdot \beta ^{(t-1)}_{j} \cdot Z^{i} \equiv \sum _{i} u_{i}\cdot Z^{i}$ is obtained. Coefficient extraction and key-switching procedures are used afterwards to obtain *n* individual L0 *TLWE* encryptions of *u*_*i*_, *i*=0…*n*−1. These L0 *TLWE* ciphertexts represent logistic regression algorithm outputs over the last iteration.

**Line 13.** The blind rotate procedure (a fundamental building block of the *TFHE* gate bootstrapping) allows to multiply a polynomial *TP*(*Z*) (called test polynomial) by *Z*^*m*^ where *m* is the message encrypted in a *TLWE* ciphertext. Moreover, the resulting *TRLWE* ciphertext noise (encrypting polynomial *TP*(*Z*)·*Z*^*m*^) is independent of the noise of input *TLWE* ciphertext encrypting *m*.

In this step of algorithm we use the blind rotate procedure *n* times to obtain *TRLWE* encryptions of $\phantom {\dot {i}\!}{TP}_{i} \cdot Z^{u_{i}}$ for *i*=0…*n*−1. Observe that ${TP}_{i} \cdot Z^{u_{i}} \equiv \sum _{j=0}^{k-1} \alpha \cdot \sigma \left (u_{i}\right)\cdot X_{i,j} \cdot Z^{j} + \ldots $ thanks to the special form of test polynomials *TP*_*i*_. Summing up these ciphertexts (i.e. obtaining $\sum _{i} {TP}_{i} \cdot Z^{u_{i}}$) and extracting the first *k* coefficients from the result we obtain *k* L2 *TLWE* ciphertexts encrypting *Δ*_*j*_, *j*=0…*k*−1.

**Line 14.** Finally, we update the L2 *TLWE* encryptions of $\beta ^{(t-1)}_{j},~j=0\ldots k-1$, by adding *e*_*j*_ and subtracting *Δ*_*j*_ obtained in previous step.

#### Step2 – incorporate *S* into the regression model

The second part of the Algorithm 2 consists of large-scale linear algebra computation in order to integrate the matrix *S* in the regression model calculated in the previous phase. We use the following input data encryptions with parameters given in Table 2:

- *X* is encrypted as (*k*+1)∗*n* individual *TRGSW* samples (level L2),

**Table 2:** Encryption parameters, also used to encrypt TRLWE or TRGSW ciphertexts

**Step**	Level	Type	n/N	**stdv**	Security	**Sample size**
1	L0	LWE	*n*=612	2^−15^	≈128	4.8 KB
	L1	RLWE	*N*=2048	2^−53^	≈128	32 KB
	L2	RLWE	*N*=8192	2^−53^	≈128	128 KB
2	L0	LWE	*n*=612	2^−15^	≫128	4.8 KB
	L1	RLWE	*N*=4096	2^−32^	≫128	32 KB
	L2	RLWE	*N*=4096	2^−48^	≫128	32 KB
	L3	RLWE	*N*=4096	2^−64^	≫128	64 KB
	L4	RLWE	*N*=4096	2^−80^	≫128	64 KB
	L5	RLWE	*N*=4096	2^−105^	≈130	64 KB
3	L0	RLWE	*N*=4096	3.2/*q* with *q*=232	≫128	32KB
	L1	RLWE	*N*=32768	3.2/*q* with *q*=2581	≈128	4.5MB
- *y* is encrypted in *nTRLWE* samples (level L2),
- *S* is encrypted as *TRGSW* matrix sample packed in slots by line in level L3.

We describe now the homomorphic evaluation of each step of Algorithm 2.

**Line 2.** We first bootstrap the probability vector to a fixed level, and we use Chimera framework described in [15] to simultaneously change the key size to support lower noise, convert *TFHE* into *HEAAN* ciphertexts, and evaluate the sigmoid function homomorphically. Although this bootstrapping takes more than 90% of the evaluation time, it has the advantage that the encryption of the database *S* is independent from the previous computations: we can iterate the initial logistic regression loop as many times as it is needed for the model to converge, which is required to support a larger number of features. We use a single coefficient per *TRLWE* ciphertext at this step (no packing).

**Line 3.** The coefficients of *S* are packed row-wise to form a matrix of *n*×(⌈*m*/4096⌉) of *TRGSW* ciphertexts. The external homomorphic product between a single-coefficient *TRLWE* ciphertext and a packed *TRGSW* ciphertext provides 4096 slots of *z*^∗^ at once. For *m*=10643 and *n*=245, we need a total of *n*×3 external products. At this depth, the *TRGSW*- *TRLWE* external product is indeed at least twice as fast as its internal *TRLWE*- *TRLWE* equivalent, whenever one of the operand is a fresh ciphertext. At this step, we support either the coefficient packing or the slot packing, depending on the encoding of the needed output.

**Line 4.** The result is a vector of *nTRLWE* ciphertext, each one holds a single coefficient $w_{i}=p_{i}-p_{i}^{2}$, each square uses one *TRLWE* internal product.

**Line 5.** We need the matrix *G* and its inverse to fulfill the algorithm. However, inverting *G* is a relatively large depth operation, so we considered two approaches: (1) we note that $G=\frac {1}{4}I_{k+1}+\varepsilon $ where the norm of *ε* is very small (unless the input dataset is exceptionnaly biased in one direction). Thus, *G*^−1^ can be approximated with its Taylor series: $4(I_{k+1}-\varepsilon +\varepsilon ^{2}-\dots)$. On the iDASH dataset, *G*^−1^=4*I*_*k*+1_ already provides a sufficient approximation. (2) the second approach consists in evaluating the Gaussian elimination loop (or better in this case, the Cholesky factorization) using one *TFHE* bootstrapping everytime a coefficient needs to be inverted. Since *G* is a very small matrix (*k*≪*n*), this step remains very fast even if an individual bootstrapping lasts a few seconds. This bootstrapping uses look-up tables to deal with the non-linearity of the inverses and simultaneously refreshes the noise of ciphertexts to the same level throughout the inversion loop. If the required precision is too large for look-up tables, we can switch to binary gates evaluating the IEEE754 floating-point division circuit. The coefficients of *G*^−1^ are computed as individual *TRLWE* ciphertexts.

**Line 6.** As in Step 3, the coefficients of *X* are stored as individual *TRGSW* ciphertexts, and the coefficients of *S* are packed row-wise (using the same ciphertext as in Step 3). The resulting *k*×*m* matrix is row-packed.

**Line 7.** Since *S* has binary entries, the element-wise product *S*⊙*S* is equal to *S*, so only the element-wise product on the right needs to be computed. If *A* is slot-packed, this hadamard product corresponds to the internal product of *TRLWE*. If *A* is coefficient packed, the squaring is merged to the next non-linear function (here, the logarithm), and handled via the *HEAAN* bootstrapping.

Depending on the choice of packing (coefficients or slots), the final output of the second phase is either (a) *z*^∗^ and *s*^∗2^ packed as slots (which can be decrypted and divided as postprocessing to reveal the t-stat), or (b) *z*^∗^ and *A* coefficient-packed, ready to pass to phase 3.

#### Step3 – stats computation in the logarithmic domain

The target outcome of the protocol is the vector of p-values of the obtained results. It must be noted that the p-values are a monotonic (but non-polynomial) function of the t-stats $\frac {\mathbf {z}^{*}_{i}}{{\mathbf {s}^{*}_{i}}^{2}}$, so they both convey the exact same information. Therefore, the computation of the p-values themselves under encryption would produce an unjustified overhead, whereas computing the t-stats and releasing them is optimal in terms of security/efficiency. Consequently, depending on the desired trade-off between accuracy and leakage, it is possible to produce the target p-values by computing the t-stats either (a) in the client-side, after decrypting (and leaking) both numerator and denominator separately, or (b) by performing a third homomorphic step that leaks only the t-stats. For option (a), the second phase outputs the slot-packed version of both numerator and denominator. Here, we detail option (b), for which we leverage on SIMD operations on *HEAAN* ciphertexts, taking as input the coefficient-packed (fully packed) versions of $\mathbf {z}^{*}_{i}$ and $\mathbf {s}^{*}_{i}$ from phase 2.

##### HEAAN-based implementation

This phase is focused on the computation of line 8 of Solution 2 in Algorithm 2, which takes as inputs the terms $\mathbf {z}^{*}_{i}$ and $\mathbf {s}^{*}_{i}$ as fully-packed level 0 RLWE encryptions. There are two options to compute the t-stats: either approximate the inverse of the denominator and multiply numerator and inverted denominator, or apply a logarithm and avoid further homomorphic products that will further reduce the precision of the results due to the approximate arithmetic used in *HEAAN*. Hence, we have chosen the second approach. This phase applies the following operations:

**1.** As the logarithm computation requires more levels than what the input encryptions can withstand, the first step is key-switching both encryptions $\mathbf {z}^{*}_{i}$ and $\mathbf {s}^{*}_{i}$ to an expanded key with higher-degree polynomials (from *N*_0_=4096 to *N*_1_=32768), resulting in (sparsely) coefficient-packed ciphertexts. At this stage, the encryptions are completely exhausted, so a bootstrapping is needed to keep computing on them.

**2.***HEAAN* boostrapping is applied to the expanded ciphers, hence achieving level 1 encryptions. After the bootstrapping, we keep slot-packing, in order to perform the coefficient-wise computation of the squaring and logarithm operations.

**3.** The numerator $\mathbf {z}^{*}_{i}$ is still a signed number, so we compute the logarithm of the absolute value by resorting to a least-squares symmetric (even-degree) polynomial approximation of the logarithm. In order to improve on the error and due to the fact that most of the relevant inputs should be far from the vertical asymptote, we use as objective function a smoothed version of the logarithm, defined as

$$ \texttt{smoothed\_logabs}(x)=\left\{\begin{array}{l}log(|x|),\quad\text{if }{|x|>th}\\ax^{2}+b,\quad\text{otherwise},\end{array}\right. $$

where *th*<1 is a pre-calculated threshold adapted to the dataset features, and *a* and *b* are computed to make the function continuous and differentiable at *x*=*th*. For the implementation, we use a degree-8 polynomial approximation of smoothed_logabs in the plausible range for the numerator values.

**4.** The denominator term $\mathbf {s}^{*}_{i}$ is first squared and centered, in order to arrive at an encryption of $\mathbf {s}^{2*}_{i}$, and then a least-squares degree-8 polynomial approximation of the smoothed logarithm is applied to the result.

**5.** The numerator is rescaled to the same quantization factor as the denominator, and both are homomorphically subtracted, hence achieving the desired encrypted result $stat_{i}=\frac {\mathbf {z^{*}}_{i}}{\sqrt {\mathbf {s^{*}}_{i}^{2}}}$, that can be sent to the client for decryption.

## Results

### Implementation details

The three parts of the homomorphic algorithm were implemented as separate applications which are executed one after another. The first application performs the logistic regression part and outputs encryptions of *X*·*β*^(*iters*)^. The second one does the generalization of the previous logistic regression model with genotype data *S*. Finally, the last application computes the quotients on the stats, that can be used to obtain the sought p-values. Besides these applications performing homomorphic computation several helper applications were implemented for generating keys, encrypting input data and decrypting the result. Data exchange (ciphertext data) between application executions is done via the file-system. That is, each application reads input data from files and writes the results to files.

C/C++ programming language was used for implementation. Two open-source HE libraries are employed: (i) *TFHE* library (https://github.com/tfhe/tfhe), in particular the torus_generic branch, optimized using AVX and FMA instructions, (ii) *HEAAN* library (https://github.com/snucrypto/HEAAN). Besides these two HE libraries, a prototype of the Chimera framework [15] was coded for the purpose of this project.

### Benchmarks and dataset details

The dataset used for the HE Track of iDASH 2018 has the following features: *m*=10643 SNPs, *n*=245 patients, *k*=3 covariates. The test environment is an Amazon T2 Xlarge VM, with 4 vCPU, 16GB memory, and 200GB of disk space. We have also tested on a m5.24xlarge VM with 96 vCPU machines to verify that the execution scales with the number of CPU without increasing the memory.

**Fig. 1:**
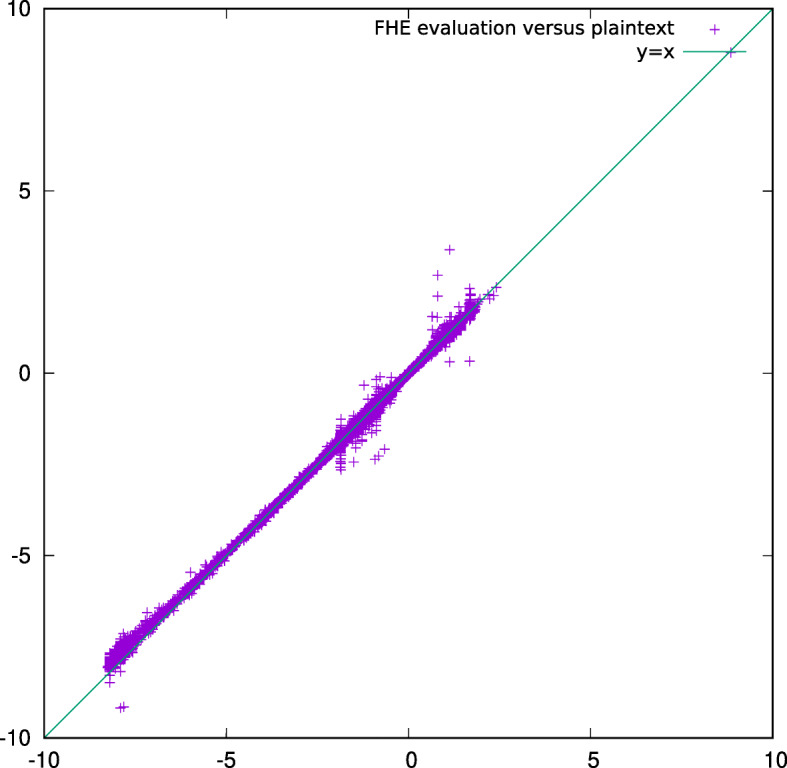
**Accuracy** Homomorphic versus plaintext computed stat vector

Table 3 shows the running time and the used memory for our solution, broken down in each of the steps. The size of the input encryptions is 5GB, including the encryption of *X*, *y* and *S*. The size of the output is 640KB including the encryption of the numerator and the denominator. The encryption parameters are given in Table 2. The numerical accuracy is depicted in Fig. 1, that plots the homomorphic versus plaintext computation of the stat coefficients.

**Table 3:** Timing and memory results

**Steps**	**Timing (4 cores)**	**Timing (96 cores)**	**RAM**
KeyGen Solution 1	5.5 mins	2.0 mins	4.4 GB
KeyGen Solution 2	6.2 mins	2.5 mins	14 GB
Encryption	7.2 mins	1.3 mins	8.6GB
Step 1	6.5 mins	0.5 mins	5.6 GB
Step 2 Bootstrapping	164 mins	3.2 mins	-
Step 2 Total	180 mins	3.5 mins	7.9 GB
Step 3	32 mins	10 mins	15 GB
Total Cloud run time Solution 1	186 mins	4 mins	7.9 GB
Total Cloud run time Solution 2	218 mins	14 mins	15 GB

## Discussion

The main part of the computation is consumed during the bootstrapping at the beginning of step 2 (more that 90% of the total evaluation time). But this bootstrapping allows us to use different values for *k* and for the number of iterations during the logistic regression phase; hence, it makes the solution much more generic than other approaches (as the depth of our circuit does not depend on the these parameters). Additionally, this is the only bootstrapped solution submitted to the iDASH competition that can be applied for different sets of parameters without re-encrypting the genomic database, making it practical for real-world applications.

One possibility to further improve the running time is to use the HEAAN bootstrapping instead of the several TFHE bootstrappings in Step 2 of the solution.

## Conclusions

The HE Track of iDash 2018 competition succeed the improvement of the actual state of art for privacy-preserving computation on genomic data. In this work we proposed fully homomorphic based solution, one of the most generic solution, that can be used with different sets of parameters. This is the first step towards the more general feature selection problem across large encrypted databases.

## Data Availability

The evaluation data can be found at http://www.humangenomeprivacy.org/2018/competition-tasks.html
